# Supplementary material for: A fault-intrusion-tolerant system and deadline-aware algorithm for scheduling scientific workflow in the cloud
Source: PeerJ Comput Sci. 2021 Nov 2;7:e747. doi: 10.7717/peerj-cs.747 (PMC8576554; doi:10.7717/peerj-cs.747)
Supplement: Supplemental Information 6 [file peerj-cs-07-747-s006.zip › Myproject1.1/docs/org/workflowsim/failure/FailureGenerator.html]

FailureGenerator


JavaScript is disabled on your browser.


- Overview
- Package
- Class
- Use
- Tree
- Deprecated
- Index
- Help

- Prev Class
- Next Class

- Frames
- No Frames

- All Classes

- Summary:
- Nested |
- Field |
- Constr |
- Method

- Detail:
- Field |
- Constr |
- Method


org.workflowsim.failure

## Class FailureGenerator

- java.lang.Object
- - org.workflowsim.failure.FailureGenerator

- ---

    

  ```
  public class FailureGenerator
  extends java.lang.Object
  ```

  FailureGenerator creates a failure when a job returns

  Since:
  :   WorkflowSim Toolkit 1.0

- - ### Constructor Summary

    Constructors

    Constructor and Description

    `FailureGenerator()`
  - ### Method Summary

    Methods

    | Modifier and Type | Method and Description |
    | --- | --- |
    | `static boolean` | `generate(Job job)` Generates a failure or not |
    | `static void` | `init()` Initialize a Failure Generator. |

    - ### Methods inherited from class java.lang.Object

      `clone, equals, finalize, getClass, hashCode, notify, notifyAll, toString, wait, wait, wait`

- - ### Constructor Detail


    - #### FailureGenerator

      ```
      public FailureGenerator()
      ```
  - ### Method Detail


    - #### init

      ```
      public static void init()
      ```

      Initialize a Failure Generator.


    - #### generate

      ```
      public static boolean generate(Job job)
      ```

      Generates a failure or not

      Parameters:
      :   `job` -

      Returns:
      :   whether it fails


- Overview
- Package
- Class
- Use
- Tree
- Deprecated
- Index
- Help

- Prev Class
- Next Class

- Frames
- No Frames

- All Classes

- Summary:
- Nested |
- Field |
- Constr |
- Method

- Detail:
- Field |
- Constr |
- Method
